# Olig2-Lineage Astrocytes: A Distinct Subtype of Astrocytes That Differs from GFAP Astrocytes

**DOI:** 10.3389/fnana.2018.00008

**Published:** 2018-02-14

**Authors:** Kouko Tatsumi, Ayami Isonishi, Miwako Yamasaki, Yoshie Kawabe, Shoko Morita-Takemura, Kazuki Nakahara, Yuki Terada, Takeaki Shinjo, Hiroaki Okuda, Tatsuhide Tanaka, Akio Wanaka

**Affiliations:** ^1^Department of Anatomy and Neuroscience, Faculty of Medicine, Nara Medical University, Kashihara, Japan; ^2^Department of Anatomy, Hokkaido University Graduate School of Medicine, Hokkaido University, Sapporo, Japan; ^3^Department of Anesthesiology, Faculty of Medicine, Nara Medical University, Kashihara, Japan; ^4^Department of Functional Anatomy, Graduate School of Medical Science, Kanazawa University, Kanazawa, Japan

**Keywords:** Olig2, GFAP, astrocyte, heterogeneity, GABA

## Abstract

Astrocytes are the most abundant glia cell type in the central nervous system (CNS), and are known to constitute heterogeneous populations that differ in their morphology, gene expression and function. Although glial fibrillary acidic protein (GFAP) is the cardinal cytological marker of CNS astrocytes, GFAP-negative astrocytes can easily be found in the adult CNS. Astrocytes are also allocated to spatially distinct regional domains during development. This regional heterogeneity suggests that they help to coordinate post-natal neural circuit formation and thereby to regulate eventual neuronal activity. Here, during lineage-tracing studies of cells expressing Olig2 using Olig2^CreER^; Rosa-CAG-LSL-eNpHR3.0-EYFP transgenic mice, we found Olig2-lineage mature astrocytes in the adult forebrain. Long-term administration of tamoxifen resulted in sufficient recombinant induction, and Olig2-lineage cells were found to be preferentially clustered in some adult brain nuclei. We then made distribution map of Olig2-lineage astrocytes in the adult mouse brain, and further compared the map with the distribution of GFAP-positive astrocytes visualized in GFAP^Cre^; Rosa-CAG-LSL-eNpHR3.0-EYFP mice. Brain regions rich in Olig2-lineage astrocytes (e.g., basal forebrain, thalamic nuclei, and deep cerebellar nuclei) tended to lack GFAP-positive astrocytes, and vice versa. Even within a single brain nucleus, Olig2-lineage astrocytes and GFAP astrocytes frequently occupied mutually exclusive territories. These findings strongly suggest that there is a subpopulation of astrocytes (Olig2-lineage astrocytes) in the adult brain, and that it differs from GFAP-positive astrocytes in its distribution pattern and perhaps also in its function. Interestingly, the brain nuclei rich in Olig2-lineage astrocytes strongly expressed GABA-transporter 3 in astrocytes and vesicular GABA transporter in neurons, suggesting that Olig2-lineage astrocytes are involved in inhibitory neuronal transmission.

## Introduction

The transcription factor Olig2 plays an essential role in the differentiation of oligodendrocytes and motor neurons in the embryonic spinal cord (Takebayashi et al., 2002; Zhou and Anderson, 2002), and in oligodendrocyte maturation in the developing ventral forebrain (Parras et al., 2007; Petryniak et al., 2007). One of the proposed major Olig2 functions is to generate oligodendrocytes by inhibiting astrocytic differentiation (Fukuda et al., 2004; Setoguchi and Kondo, 2004). In the last decade, a number of genetic fate-mapping studies using Olig2^CreER^ mice have demonstrated that Olig2-positive cells (Olig2 cells) generate oligodendrocytes, astrocytes and neurons in the developing brain, and their differentiation properties vary in a brain region-specific manner (Miyoshi et al., 2007; Ono et al., 2008). These detailed studies support the concept that Olig2 cells form heterogeneous progenitor pools in the developing forebrain (Ono et al., 2008). In the adult brain, most Olig2 cells co-express NG2 proteoglycan and are termed adult oligodendrocyte progenitor cells (OPCs) (Goldman, 2003). They generate mainly OPCs/NG2 glia or mature oligodendrocytes and a subset of astrocytes, but not neurons (Dimou et al., 2008; Tatsumi et al., 2016). In injured or pathological states of the adult brain, Olig2 cells generate OPCs/NG2 glia, mature oligodendrocytes and a portion of reactive astrocytes, but, again, not neurons (Magnus et al., 2008; Tatsumi et al., 2008; Islam et al., 2009; Zhao et al., 2009; Guo et al., 2011; Shimizu et al., 2013). These genetic fate-mapping studies indicate that Olig2 cells mainly generate oligodendrocyte-lineage cells throughout life, while neurogenesis is limited in the embryonic stage and never occurs in the post-natal brain. The role of Olig2 in astrogliogenesis is much less well understood, because it has received little attention so far.

Recently, we found that Olig2-lineage astrocytes clustered in specific brain nuclei in the adult mouse (Tatsumi et al., 2016). During lineage-tracing studies of cells expressing Olig2 in Olig2^CreER^; ROSA-GAP43-EGFP mice (Tatsumi et al., 2008), we found that Olig2-lineage mature astrocytes preferentially cluster in the globus pallidus, which exerts pivotal functions in the indirect pathway of the basal ganglionic circuit. These experiments took advantage of the GAP43-EGFP fusion protein, whose targeted distribution to the plasma membrane allowed us to visualize cellular morphology in detail (e.g., the shapes of fine processes). GFP immunoelectron microscopy illustrated that immunoreactive fine processes elaborated arborization into the neuropil and attached to synaptic sites. Morphometric analyses revealed that astrocytic fine processes of the Olig2-lineage astrocytes underwent plastic changes that correlated with overall running activity, suggesting that they actively modulate motor functions (Tatsumi et al., 2016). In addition, we recently noticed that another reporter line (Ai39 mouse; see Materials and Methods) was very potent in illustrating detailed morphology of a recombined cell.

Neurotransmitter uptake is one of the important functions of astrocytes (Haydon, 2001). In the vast amount of brain excitatory synapses, astrocytes uptake excess amount of glutamate from synaptic clefts through glutamate transporters such as GLT-1 and GLAST (Chaudhry et al., 1995; Rothstein et al., 1996; Tanaka et al., 1997). Another important function of astrocytes is gliotransmission, although its functional significance is recently in dispute (Fiacco and McCarthy, 2018; Savtchouk and Volterra, 2018). For example, G-protein coupled receptor-regulated glutamate exocytosis has been demonstrated (Cali et al., 2014). In addition to the glutamate transporters, astrocytes are also known to express GABA transporter, GAT-3 (Fraser et al., 1994; Durkin et al., 1995). GAT-3 is mainly found in the retina, olfactory bulb (OB), and globus pallidus (Minelli et al., 1996; Jin et al., 2011a,b). In the basal ganglia, GAT-3 modulates neuronal activities in concert with another GABA transporter, GAT-1, which is primarily expressed in neurons (Kirmse et al., 2009; Galvan et al., 2010; Jin et al., 2011a). As in the case for gliotransmission of glutamate, GABA is also released from astrocytes through bestrophin channel (Lee et al., 2010). These findings imply that a subgroup of astrocytes may be subsidiary to inhibitory neuronal circuits by transporting GABA from and/or releasing GABA to synaptic clefts.

The fact that Olig2-lineage astrocytes clustered in specific brain nuclei prompted us to construct a distribution map of Olig2-lineage astrocytes in the whole brain. We also performed similar genetic marking of glial fibrillary acidic protein (GFAP)-positive astrocytes, and then compared the distribution pattern of Olig2-lineage astrocytes with that of GFAP-positive astrocytes. We found that these two populations of astrocytes were differentially distributed in the adult mouse brain. Olig2-lineage astrocytes tended to express GABA transporter-3 (GAT-3), and their fine processes frequently attached to synapses with vesicular GABA transporter-positive axon terminals. Based on the above data, we discuss possible functional implications of Olig2-lineage astrocytes.

## Materials and methods

### Animals

We crossed Olig2 knock-in mice (Olig2^KICreER^) (Takebayashi et al., 2002) or GFAP^Cre^ mice (Garcia et al., 2004; Herrmann et al., 2008) with reporter mice Rosa-CAG-LSL-eNpHR3.0-EYFP (Ai39 mice, Jackson Laboratory, Stock No: 014539) to obtain Olig2^KICreER/WT^; Ai39 mice (hereafter termed Olig2^CreER^- Ai39 mice) and GFAP^KICre^; Ai39 mice (hereafter termed GFAP^Cre^-Ai39 mice). In Ai39 mice, cre-inducible halorhodopsin (eNpHR3.0) fused with YFP at its C-terminus is driven by a specially designed expression cassette in a modified *Rosa26* locus, which showed efficient and strong YFP fluorescence at the membrane (Madisen et al., 2009, 2012). The YFP is fused to the C-terminus of the halorhodopsin and is localized in the cytoplasmic side. It clearly revealed cellular morphology without YFP immunostaining. In the present study, we used theses double transgenic mice for immunofluorescence study.

We also crossed Olig2 knock-in mice (Olig2^KICreER^) and ROSA-GAP43-EGFP reporter line (Soriano, 1999) to obtain Olig2^KICreER/WT^; GAP43-EGFP mice (hereafter termed Olig2^CreER^-GFP mice). This reporter mice were particularly useful for visualizing the morphology of cells that underwent recombination, because GAP43-EGFP is a fusion protein between GFP and the N-terminus (amino acids 1–20) of GAP-43, which has been implicated in plasma membrane targeting through palmitoylation (Liang et al., 2002). Although the fluorescence of the GAP43-EGFP was generally weaker than that of the Ai39 reporter line, the moderate amount of the fusion protein was suitable for immunoelectron microscopic detection. Taking these experimental conditions into account, we employed Olig2^CreER^-, and GFAP^Cre^-Ai39 mice for fluorescence microscopy and Olig2^CreER^-GFP mice were used for electron microscopy.

All the mice were maintained in a mixed genetic background; Olig2^CreER^-Ai39 mice (C57BL/6 and 129 S6/Sv strains), GFAP^Cre^-Ai39 mice (C57BL/6 and FVB/N strains) and Olig2^CreER^-GFP mice (C57BL/6 and 129 X1/svJ strains). They were housed in standard cages under a 12 h light/dark cycle and temperature-controlled conditions. In the present study, we used 3–5 mice for each transgenic mouse strain and all the mice were 11 weeks old. All the protocols for the animal experiments were approved by the Animal Care Committee of Nara Medical University in accordance with the policies established in the NIH Guide for the Care and Use of Laboratory Animals.

### Tamoxifen administration

**Figure 1:**
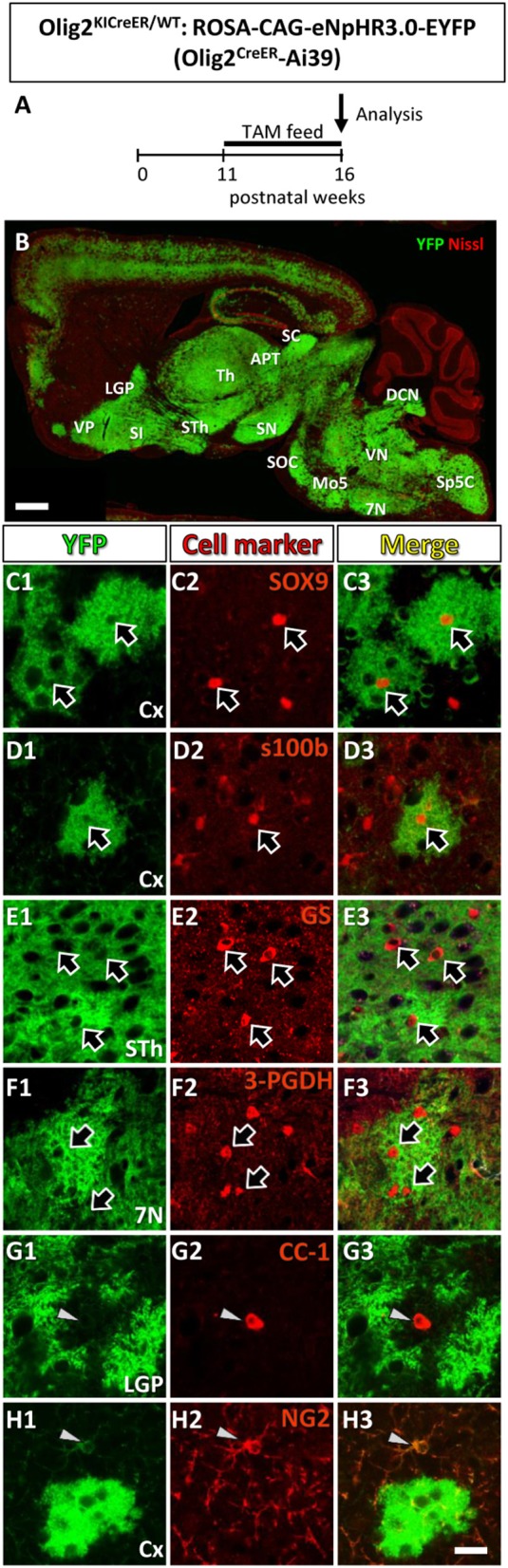
Olig2-lineage cells in the adult Olig2^CreER^-EYFP mice express astrocytic marker. **(A)** The experimental schedule of tamoxifen administration was depicted. Tamoxifen was orally administered (TAM feed) for 5 consecutive weeks and at the end of the 16th week, mice were sacrificed and subjected to analyses. **(B)** Low-magnification view of YFP-positive Olig2-lineage cells (green) and Nissl stain (red) in a sagittal brain section of Olig2^CreER^-YFP mice. **(C–H)** Double immunofluorescence with cell marker antibodies showed that Olig2-lineage bushy cells were positive for mature astrocytic marker: SOX9 (**C1–C3**; arrows), s100β (**D1–D3**; arrow), GS (**E1–E3**; arrows), 3-PGDH (**F1–F3**; arrows) in brain regions indicated. They were negative for mature oligodendrocyte marker, CC-1 (**G1–G3**; arrowhead), and NG2 (OPCs/NG2 glia marker, **H1–H3;** arrowheads) in their nuclei (**G,H**; arrows). LGP, lateral globus pallidus; VP, ventral pallidum; SI, substantia innominata; Th, thalamus; STh, subthalamic nucleus; ZI, zona incerta; APT, anterior pretectal nucleus; SC, superior colliculus; SN, substantia nigra; SOC, superior olivary complex; Mo5, motor trigeminal nucleus; DCN, deep cerebellar nuclei; VN, vestibular nuclei; 7N, facial nucleus; Sp5, spinal trigeminal nucleus. Scale bar: 1 mm **(B)**, 20 μm (**H3**, for **C–H**).

**Figure 2:**
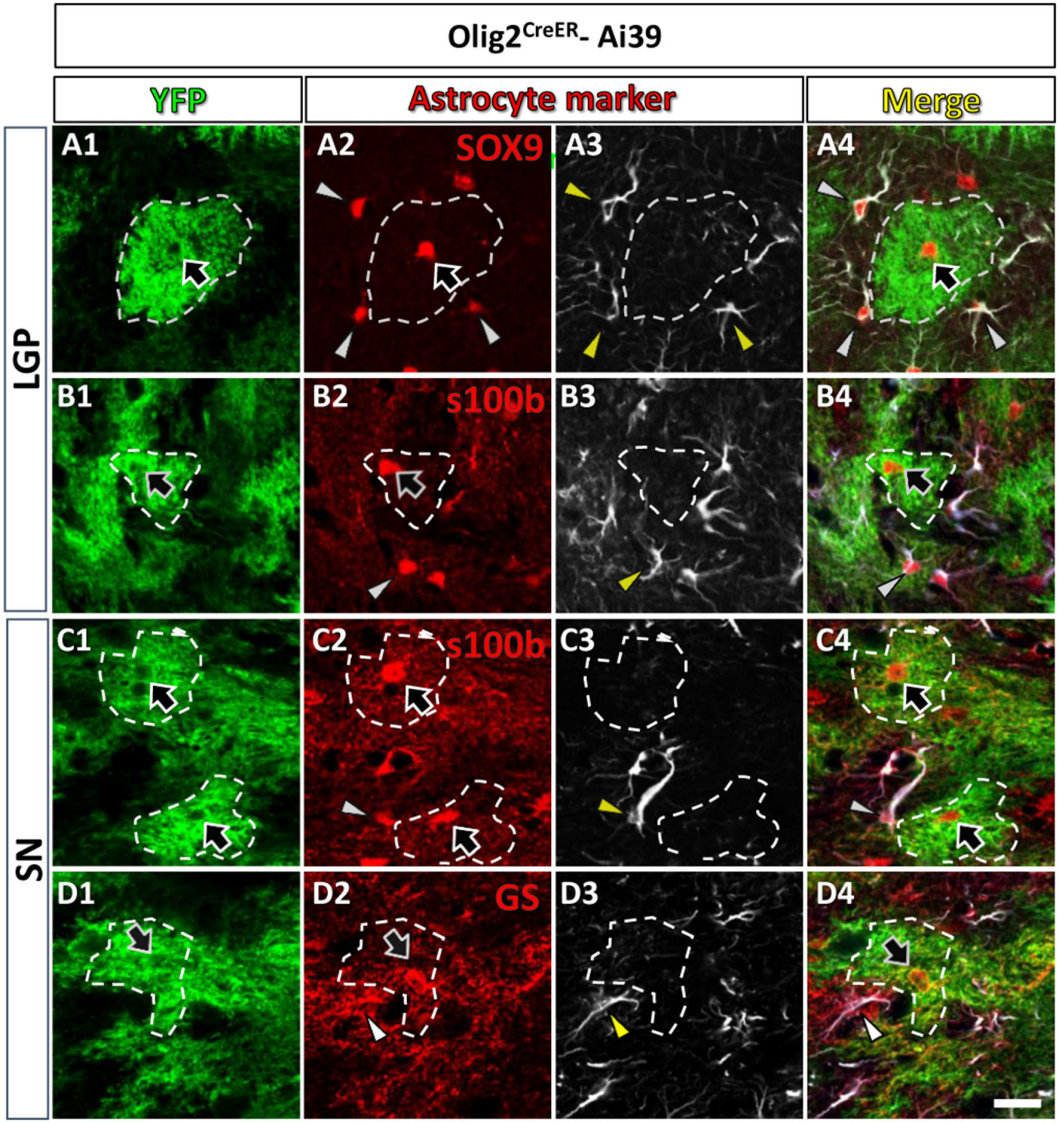
Olig2-AS expressed low level of GFAP in the LGP and the SN. Olig2-AS co-expressed mature astrocytic marker SOX9 (arrow, **A2**), s100β (arrows, **B2,C2**) and GS (arrow, **D2**) in the LGP **(A,B)** and the SN **(C,D)**. GFAP immunoreactive structures (arrowheads, **A3–D3**) were barely detected in the Olig2-AS territories (bordered with white dotted lines). Note that mature astrocyte markers (SOX9, s100β, and GS, arrowheads in **A2–D2**) were co-expressed with GFAP immunoreactivities (arrowheads in **A4–D4**). Scale bar (**D5**, for all panels): 20 μm.

In the present study, we employed oral administration of tamoxifen. Tamoxifen (TM, Sigma Aldrich, Japan) was mixed with powdered chow (0.5 mg/g normal chow). Olig2^CreER^-Ai39 mice for immunofluorescence experiments were allowed to access the tamoxifen-containing chow *ad libitum* from 11 to 16 weeks old (see Figure 1A). This oral administration method is convenient for continuous administration and results in efficient induction of recombination while minimizing stress on the mice (Casanova et al., 2002; Kiermayer et al., 2007; Feil et al., 2009; Welle et al., 2009). Olig2^CreER^-GFP mice for immunoelectron microscopy were also fed with tamoxifen-containing chow as described above (see Figure 2A). All the mice appeared in healthy condition, although some mice were lost up to 10% of their body weight during the tamoxifen-fed period.

### Tissue preparation and immunofluorescence experiments

Mice were perfused with 4% paraformaldehyde (PFA) in phosphate-buffered saline (PBS). Brains were removed and post-fixed in 4% PFA, and cryopreserved in 30% sucrose until they sank, and cryosections were cut at 30-μm thickness on a cryostat (Leica CM1860, Leica Microsystems, Japan). Immunofluorescence experiment was performed as described previously (Tatsumi et al., 2016). The primary antibodies were listed in the Table 1. Alexa-594- or 647-conjugated antibodies (1:1,000, Jackson ImmunoResearch, USA) were used as secondary antibodies. Blue-fluorescent Nissl stain (1:200, Neurotrace 435/455, ThermoFisher Scientific, Japan) was used as a counterstaining.

**Table 1:** List of primary antibodies used.

**Primary antibodies**	**Source**	**Identifier**	**Dilution**
**FOR IMMUNOFLUORESCENT STAINING**			
Rabbit anti-GFAP	Dako	Z0334	1:2,000
Mouse anti-GFAP	EMD millipore	MAB360	1:500
Rabbit anti-S100β	Abcam, Japan	AB41548	1:5,000
Rabbit anti-3-PGDH	Frontiers Institute	Af303	1:6,000
Mouse anti-GS	EMD millipore	MAB302	1:2,000
Goat anti-SOX9	R&D systems	#P48436	1:1,000
Rabbit anti-GLAST	Cell signaling	#5684	1:1,000
Rabbit anti-GLT-1	Cell signaling	#3838	1:1,000
Rabbit anti-GAT-3	EMD millipore	AB1574	1:200
Guinea pig anti-GAT-3	Synaptic systems	Cat. No. 274301	1:2,000
Rabbit anti-VGAT	Synaptic systems	Cat. No. 131013	1:200
Guinea pig anti-VGIuT1	Miyazaki et al., 2003	RRID: AB_2571618	1 μg/ml
Guinea pig anti-VGluT2	Miyazaki et al., 2003	RRID: AB_2571621	1 μg/ml
Rabbit anti-GFP	Takasaki et al., 2010	RRID: AB_2571573	3 μg/ml
**FOR IMMUNOELECTRON MICROSCOPY**			
Rat anti-GFP	Nacalai tesque	GF090R	1:500
Rabbit anti-GFP	Takasaki et al., 2010	RRID: AB_2571573	1 μg/ml
Rabbit anti-VGAT	Synaptic systems	Cat. No.l31013	1:3,000
Guinea pig anti-VGluTl	Miyazaki et al., 2003	RRID: AB_2571618	1 μg/ml
Guinea pig anti-VGluT2	Miyazaki et al., 2003	RRID: AB_2571621	1 μg/ml
Guinea pig anti-VIAAT	Miyazaki et al., 2003	RRID: AB_2571624	1 μg/ml

**Figure 3:**
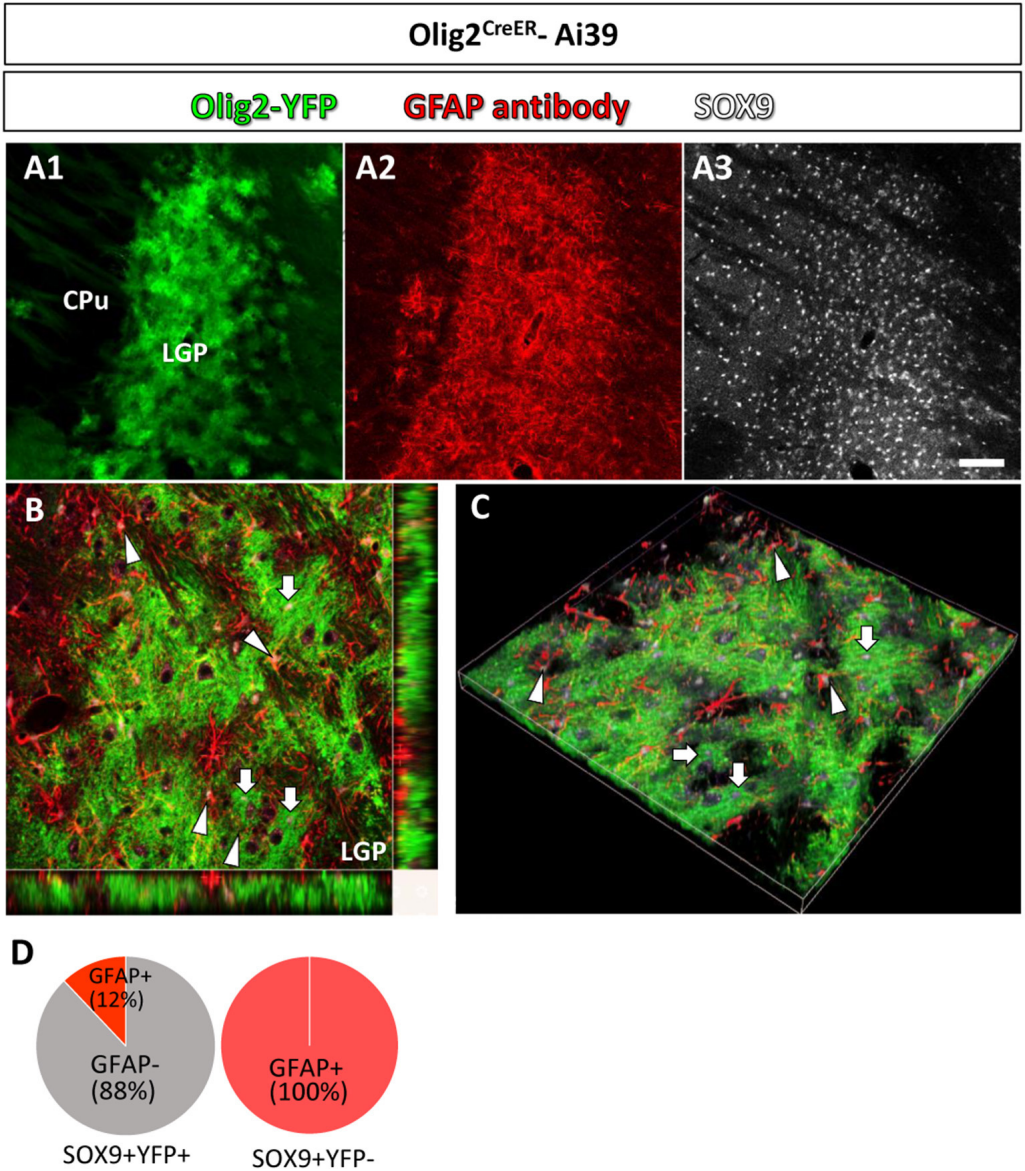
Olig2-AS and GFAP-immunoreactive astrocytes show differential localization in a single brain nucleus. In a low-magnification view, Olig2-AS (in green fluorescence) and GFAP-immunolabeled astrocytes (in red fluorescence) were densely localized in the LGP (**A1,A2**, respectively**)** and SOX9 immunoreactive astrocytes were more widely distributed in CPu and LGP **(A3)**. **(B)** The orthogonal views (x-y, y-z, and x-z planes) revealed that SOX9 positive Olig2-AS (arrows) and SOX9 positive GFAP-expressing astrocytes (arrowheads) occupied adjacent but non-overlapping territories in the LGP. **(C)** The z-stack 3D images of the same area of **(B)**. **(D)** Semi-quantitative analyses revealed that 88% (204/232, *n* = 3) of SOX9-positive YFP-positive Olig2-AS were GFAP-negative and 12% (28/232, *n* = 3) of them co-expressed GFAP. All the SOX9-positive YFP-negative cells in the LGP were positive for GFAP (281/281, *n* = 3). Scale bar (**A3**, for **A1,A2**): 100 μm.

All the fluorescence images were obtained with a confocal laser scanning microscope (Nikon C2-NiE, Tokyo, Japan) as single planes or as z-stack projections (x-axis by y-axis, 1,024 by 1,024 pixels). For wide-field fluorescence images (e.g., half coronal section or whole sagittal section of mouse brain), we captured 50~80 images for whole area with 10 × objective lens (NA 0.45, UpanApo, Nikon) at 1.5 optical zoom by motorized x/y stage, and images were tiled to construct a large image. We obtained the z-stack multi-channel images at 1 μm intervals with 20 × objective lens (NA 0.75, UpanApo, Nikon) for the globus pallidus and the images generated orthogonal views (x-z and y-z planes) and a 3D rendering view (see Figures 3B,C). For high magnification images, we used 60 × water-immersion objective lens (NA 1.45, UpanApo, Nikon) at 1.5 optical zoom. All the images were processed by Nikon Nis-Elements software (version 4.11).

**Figure 4:**
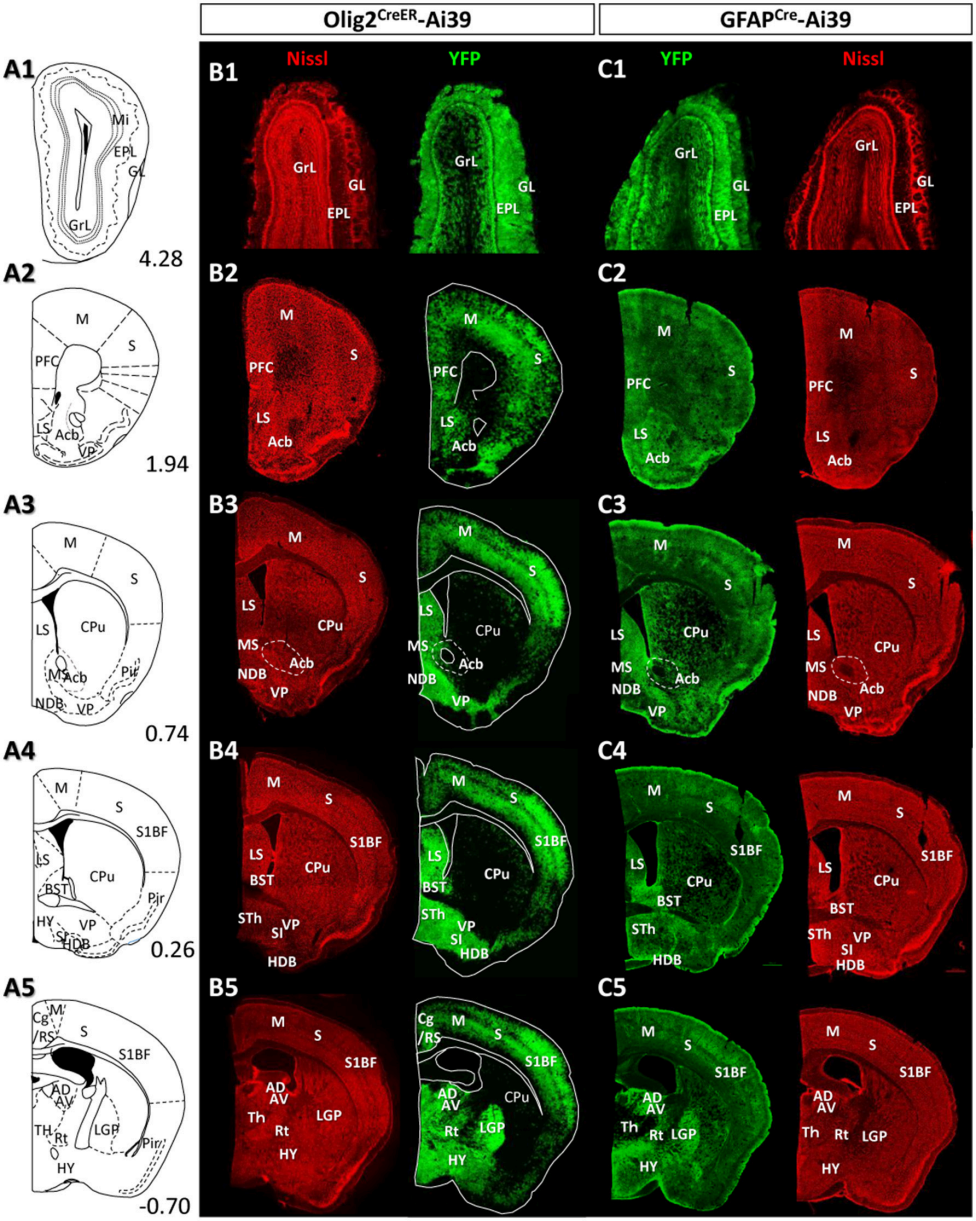
Distribution patterns of Olig2-AS and GFAP-AS in the adult Olig2^CreER^- and GFAP^Cre^-Ai39 mice (olfactory bulb to forebrain). Illustrated brain sections with the names of brain nuclei are shown in a rostro-caudal order **(A1–A5)**. The numerical values in the lower right corners indicate distance (mm) from the bregma. All the abbreviations of brain nuclei are according to the Mouse Brain Atlas in Stereotaxic Coordinates (Franklin and Paxinos, 1997) and are listed at the end of the main text. The distributions of Olig2- **(B1–B5)** and GFAP-AS **(C1–C5)** were compared in coronal sections of the same rostro-caudal position of Olig2^CreER^- and GFAP^Cre^-Ai39 mice, respectively. All coronal sections were counterstained by Nissl, and identified brain nuclei are indicated in each section.

The YFP intensity of each brain region in the low magnification images was measured using Nikon Nis-Elements software. To standardize the fluorescence intensities among different sections, the value of fluorescence intensity was divided by the value of background intensity, yielding a ratio. For objective evaluation of regional fluorescence, a researcher who was blind to the nature of an image performed measurements of regional fluorescence with the software and calculate the brain region/background ratios. We then classified these ratios into three groups (strong; larger than 20, moderate; 10–20, weak/absent; less than 10) and the groups were color-coded (strong; red, moderate; yellow, weak/absent; blue) for Table 2. For example, the panel B4 in the Figure 4 contained the regions of strong fluorescence such as the lateral septum (LS; 22.5) and the somatosensory cortex (S1BF; 28.6), the region of moderate fluorescence such as the motor cortex (M, 14.6) and the regions of weak or absent fluorescence such as the caudate putamen (CPu, 2.2). In the Table 2, it should be noted that the color-coded ratios were relative among the sections of each double transgenic line (i.e., Olig2^CreER^-Ai39 or GFAP^Cre^-Ai39 mice) and could not be directly compared.

**Table 2:** Distribution of Olig2-AS and GFAP-AS in the adult mouse brain.

	**Olig2^CreER^**	**GFAP^Cre^**
**CEREBRUM**		
**Olfactory bulb (OB)**		**Immuno**
Glomerular layer (GL)		
External plexiform layer (EPL)		
Mitral cell layer (Mi)		
Inner plexiform layer (IPL)		
Granular cell layer (GrL)		
**Striatum (STR)**		
Caudla putamen (Cpu)		
Accumbens nucleus (Acb)		
Lateral septal nucleus, dorsal part (LSD)		
Lateral septal nucleus, intermediate (LSI)		
Septofimbrial nucleus (SFi)		
**Pallidum (PAL)**		
Lateral globus pallidus (LGP)		
Medial globus pallidus (MGP)		
Ventral pallidum (VP)		
Substantia innominata (SI)		
Magnocellular preoptic nucleus (MCPO)		
Medial septum nucleus (MS)		
Diagonal band nucleus (NDB)		
Bed nucleus of the stria terminals (BST)		
**Hippocampal region (HIP)**		**Immuno**
CA1 pyramidal layer (PyCA1)		
CA1 others		
CA2 pyramidal layer (PyCA2)		
CA2 others		
CA3 fields		
Dentate gyrus, molecular layer (molDG)		
Dentate gyrus, granule cell layer (GrDG)		
Dentate gyrus, polymorph layer (PoDG)		
**Thalamus**		
Anterodorsal, anteroventralthalamic nucleus (AD, AV)		
Reticular thalamic nucleus (Rt)		
Thalamic nucleus (MD, CM, AM, VA, PV, VPL, VPM, PF)		
Thalamic nucleus (Po, LP)		
Geniculate nucleus (MG, VLG)		
Periventricular fiber system (pv)		
Medial habenular nucleus (MHb)		
Lateral habenular nucleus (LHb)		
**Hypothalamus**		
Preoptic area (LPO, MPA, MPO)		
Lateral hypothalamic nucleaus (LH)		
Hypothalamic area (AH, PH)		
Paraventricular zone (PaV, Papo, Spa, SCh)		
Subthalamic nucleus (STh)		
Zona incerta (ZI)		
Mammilary body (MM)		
**Cerebral cortex (CTX)**		
Somatosensory area (S1, S1BF, S2)		
Prefrontal cortex (PFC)		
Motor area (M1, M2)		
Cingulate retrosplenial cortex (Cg/RS)		
Visual area (V1)		
Visual area (V2)		
Amygdalohipocampal area (Ahi)		
Basomedial amygdaloid nucleusposterior part (BMP)		
Posterior cortical amygaloid nucleus (PMCo)		
Retrosplenial agranular cortex (RSA)		
Retrosplenial granular (RSG)		
Others		
**MIDBRAIN**		
Superior coliculus (SC)		
Inferior colliculus (IC)		
Pretectal nucleus (APT, OPT, PPT, MPT)		
Deep mesencephalic nucleus (DpMe)		
Red nucleus (R)		
Periaqueductal gray (PAG)		
Ventral tegmental area (VTA)		
Substantia nigra (SN)		
**PONS**		
Principal sensory trigeminal nucleus (Pr5)		
Motor trigeminal nucleus (Mo5)		
Supratrigerminal nucleus (Su5)		
Superior olivary nuclei (SOn)		
Pontine nuclei (Pn)		
**MEDULLA**		
Vestibular nuclei (MV, LV, SpVe, SuVe)		
Spinal trigeminal nucleus (Sp5)		
Facial nucleus (7N)		
Gigantocellular reticular nucleus (Gi)		
Intyermediate reticular nucleus (IRt)		
Parvicellular reticular nucleus (PCR)		
**CEREBELLUM**		
Deep cerebellar nuclei (DCN)		
Vestibulocerebellar nucleus (VeCb)		
		
Cerebellar cortex (CbX)		
		Strong
		Moderate
		Weak/absent

*Staining intensities were arbitrarily classified into three categories (Strong, moderate, and weak/absent).The intensity of each brain nucleus was evaluated by a person who did not know the nature of the staining. Note that GFAP immunoreactivity instead of GFAP-YFP expression was employed in the case of the olfactory bulb and hippocampal regions. Abbreviations of brain nuclei are according to the Mouse Brain Atlas in Stereotaxic Coordinates (Franklin and Paxinos, 1997)*.

### Immunoelectron microscopy

Pre-embedding double immunoelectron microscopy was performed as described previously with a few modifications (Yamasaki et al., 2010). Vibratome sections were incubated in blocking solution (Aurion, Netherlands) for 30 min and then with a cocktail of primary antibodies (for GABAergic terminals; rabbit anti-GFP antibody and guinea pig anti-VGAT antibody, for glutamatergic terminals; rabbit anti-GFP antibody and guinea pig anti-VGluT1/2 antibodies) diluted with 1% BSA/ 0.002% saponin/PBS overnight. The sections were washed with 0.002% saponin/ PBS, incubated with 1.4 nm gold particles conjugated anti-guinea pig IgG (1:100, Nanoprobes, Stony Brook, NY) for 4 h. After washing, immunogold particles were intensified using a silver enhancement kit (R-Gent SE-EM; Aurion, Netherlands) for 60 min, washed, and incubated with peroxidase labeled anti-rabbit IgG (414341F, Nichirei Bioscience, Japan) for 4 h and visualized with chromogenic reaction of 3,3′-diaminozidine (DAB). Sections were further treated with 1% osmium tetroxide for 15 min on ice, stained by 2% uranyl acetate for 15 min.

In addition to the method described above, we performed another method of double immunoelectron microscopy. Briefly, vibratome sections were incubated a cocktail of primary antibodies (for GABAergic terminals; rat anti-GFP antibody and rabbit anti-VGAT antibody, for glutamatergic terminals; rat anti-GFP antibody and guinea pig anti-VGluT1/2 antibodies) diluted with 1% BSA/0.002% saponin/PBS overnight. The sections were washed, incubated with peroxidase-labeled anti-rat IgG (414311F, Nichirei Bioscience, Japan), and followed by DAB chromogenic reaction for GFP visualization. After washing, the sections were incubated either with biotinylated anti-rabbit antibody (BA-1000, Vector Laboratories, Japan, for GABA) or biotinylated anti-guinea pig antibody (BA-7000, Vector Laboratories, Japan, for glutamate), followed with peroxidase-labeled streptavidin (426061, Nichirei Bioscience, Japan) incubation, and then subjected to DAB chromogenic reaction again to visualize axon terminals with synaptic vesicles (VGAT or VGluT1/2 positive). Then, sections were treated with 1% osmium tetroxide for 60 min.

Although the latter DAB-DAB combination method yielded results comparable to those with immunogold-DAB method (see **Figure 7**), we found that the outlines of the DAB-labeled axon terminals were much easier to observe than those of the nanogold-labeled ones. We therefore employed the DAB combination method in semi-quantitative analyses of association between axon terminals and astrocytic fine processes (see below).

All the sections were dehydrated through a graded ethanol series, and flat-embedded in epoxy resin (Epon812 resin embedding kit, TAAB Laboratories Equipment, Japan). The resin was polymerized for 2 days at 60°C. We then carefully dissected out regions containing well-isolated GFP-positive bushy cells from a flat-embedded section under a light microscope (a representative image, inset in Figure 2B). The semi thin (1 μm) and ultrathin (70–80 nm) sections were cut on an ultramicrotome (Leica EM UC7, Leica Microsystems, Japan). The ultrathin sections were collected onto formvar-coated single whole grid, and observed under a JEOL transmission electron microscope (JEM-1400plus, JEOL, Japan).

In semi-quantitative analyses of association between axon terminals and astrocytic fine processes, a researcher took electron microscopic images that contained immunoreactive axon terminals (either VGAT-positive or VGluT1/2-positive) and astrocytic fine processes. Another researcher who was blind to the nature of vesicle transporter immunoreactions counted the numbers of immunoreactive terminals attached with astrocytic fine processes and the numbers of immunoreactive terminals without process attachments in the images. The counts were later summed and subjected to statistical analyses.

## Results

### The adult mouse brain contains Olig2-lineage astrocytes

Figure 1A showed the schedule of oral tamoxifen administration for Olig2^CreER^-Ai39 mice for immunofluorescence study. As shown in Figure 1B, continuous feeding with tamoxifen-containing chow markedly induced Cre-mediated recombination, yielding robust YFP labeling of Olig2-lineage cells. We previously reported that there were two types of Olig2-lineage cells in the adult brain (Tatsumi et al., 2008, 2016). The cell type with strong YFP fluorescence and bushy morphology were mature astrocytes (Tatsumi et al., 2016), and we confirmed that the bushy cells were positive for mature astrocyte markers in various brain regions, such as SOX9, S100β (Figures 1C1–C3, D1–D3, Figure S1) in the cerebral cortex, 3-PGDH (Figures 1E1–E3, Figure S1) in the facial nucleus (7N), GS (Figures 1F1–F3, Figure S1) in the subthalamic nucleus. The subcellular localization patterns varied among the mature markers; transcription factor SOX9 was found in the nucleus (Figure S1D), while the enzyme GS was primarily present in the cytoplasm, but was found little in the distal processes (Figure S1B). It should be noted that GS was distributed to the fine processes of Olig2-AS in addition to the cytoplasm in the cerebral cortex (Figure S1B; cx), but not in the other brain regions such as the globus pallidus or thalamus (Figure S1B; LGP, Th). The mechanisms underlying the differential subcellular distribution are unclear. The other type of YFP-positive cells was of oligodendrocyte lineage, including OPCs. We observed that mature oligodendrocyte marker CC-1 was positive in some of the YFP-expressing cells (Figures 1G1–G3, Figure S1), and immature oligodendrocyte marker NG2 was positive in other YFP-expressing cells (Figures 1H1–H3, Figure S1). The overall morphologies of these oligodendrocyte-lineage cells were clearly distinct from that of the bushy cells and these cells could not be detected in low magnification view, unless they were immunolabeled with anti-YFP antibody. This was most likely because polydendric processes were much simpler than bushy processes and the membrane-targeted YFP was less distributed in the oligodendrocyte-lineage cells. Taken together, the YFP-positive cells observed in low magnification view (Figure 1B) likely reflect the Olig2-lineage astrocytes.

### Olig2-AS express low level of GFAP

As mentioned above, Olig2-AS expressed several astrocytic markers (arrows, Figures 1, 2), but we noticed that Olig2-AS expressed low level of GFAP, the cardinal astrocytic marker. To confirm this notion, we performed double immunofluorescence study with YFP and various astrocytic markers including GFAP in Olig2^CreER^-Ai39 mice. Since GFAP immunoreactivity is not uniformly detected in the whole brain, we selected two regions, the lateral globus pallidus (LGP) and the substantia nigra (SN), where a number of GFAP-positive astrocytes exist. GFAP-positive cells co-expressed astrocytic markers such as SOX9 (arrowheads, Figures 2A2–A4), S100β (arrowheads, Figures 2B2–4,C2–C4) and GS (arrowheads, Figures 2D2–D4). On the other hand, Olig2-AS with bushy morphology also expressed astrocytic markers such as SOX9 (arrows, Figures 2A2,A4), S100β (arrows, Figures 2B2,B4,C2,C4) and GS (arrows, Figures 2D2,D4). We delineated territories of Olig2-AS with dotted line in the Figure 2 and even in the presence of GFAP-positive astrocytes, the Olig2-AS seldom overlapped with the GFAP-positive structures (Figures 2A3,B3,C3,D3).

In a low magnification view, Olig2-AS (YFP positive) were densely distributed in the LGP (Figure 3A1) and GFAP immunoreactivity was also strong (Figure 3A2). SOX9 positive cells were more widely observed in the LGP and the CPu (Figure 3A3). The orthogonal views (Figure 3B) and z-stack 3D images (Figure 3C) obtained with a confocal laser scanning microscope revealed that SOX9 positive Olig2-AS (arrows, Figures 3B,C) and SOX9 positive GFAP-positive astrocytes (arrowheads, Figures 3B,C) occupied adjacent but non-overlapping territories in the LGP. Semi-quantitative analyses revealed that 88% (204/232, *n* = 3) of SOX9-positive Olig2-AS were GFAP-negative and 12% (28/232, *n* = 3) of them co-expressed GFAP (Figure 3D). All of the SOX9-positive YFP-negative cells in the LGP co-expressed GFAP (281/281, *n* = 3) (Figure 3D). These findings illustrated that Olig2-AS constitute a sub-population of astrocytes distinct from GFAP-positive astrocytes.

### The distribution of Olig2-AS in the adult brain differs from that of GFAP-AS

**Figure 5:**
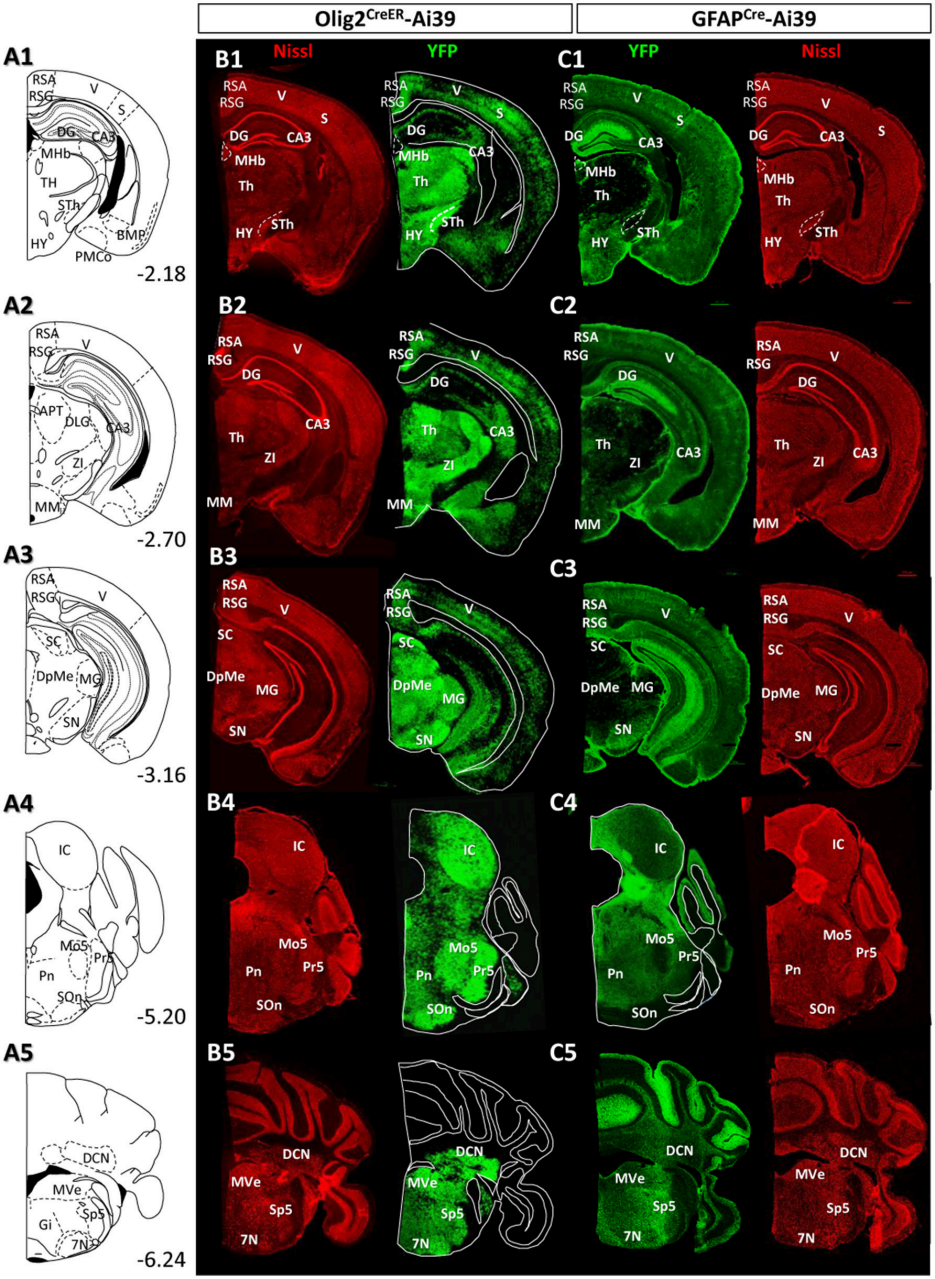
Distribution patterns of Olig2-AS and GFAP-AS in the adult Olig2^CreER^- and GFAP^Cre^-Ai39 mice (forebrain to brainstem). The brain images, corresponding illustrations **(A1–A5)**, coronal images of Olig2^CreER^ **(B1–B5)** and GFAP^Cre^-Ai39 mice **(C1–C5)** with names of brain nuclei are presented as in this figure. Sections more caudal to those in this Figure 4 are shown.

Given the clear contrast in distribution patterns of Olig2-AS and GFAP-positive astrocytes, we mapped distribution of GFAP-positive astrocytes in the brain of GFAP^Cre^-Ai39 mice and compared it with that of Olig2-AS. The GFAP^Cre^-Ai39 mice showed strong YFP fluorescence in recombinant cells and the strong labeling was suitable for comparison with the strong fluorescence of Olig2-AS visualized after continuous oral administration of tamoxifen (see above). There is, however, a caveat: some of the recombinant cells may not be astrocytes but neurons in specific brain regions such as the OB and hippocampus, where neurogenesis takes place from GFAP-positive neuronal stem cells (Kempermann et al., 1997; Eriksson et al., 1998; Seri et al., 2001). In these regions, we additionally performed GFAP immunofluorescence to distinguish neurons from astrocytes (Figures S2A,B), since neurons quickly lose GFAP protein expression as they mature while astrocytes retain GFAP expression. The genetically labeled astrocytes in the GFAP^Cre^-Ai39 mice are hereafter referred to as GFAP-astrocytes (GFAP-AS). Figures 4, 5 show low-magnification images of coronal brain sections of the same rostro-caudal position of both Olig2^CreER−^Ai39 mice and GFAP^Cre^-Ai39 mice. As a reference for brain nuclei, we illustrated the corresponding section images (Figures 4A, 5A) and the borders of anatomical regions (Figures 4B,C, 5B,C) based on the atlas image according to the mouse brain map in stereotaxic coordinates (Franklin and Paxinos, 1997). In the following sections, we focus on the major brain regions where a significant difference was observed between Olig2-AS and GFAP-AS distribution. Regarding the densities of labeled cells in each brain region, we applied arbitrary criteria (strong, moderate and low/absent) (for classification details, see Materials and Methods) and summarize the distribution patterns of Olig2-AS and GFAP-AS in the Table 2.

#### Olfactory bulb

It is well known that the OB has a multi-layered cellular architecture; these are the glomerular layer (GL), external plexiform layer (EPL), mitral cell layer (Mi), internal plexiform layer (IPL), and granule cell layer (GrL) in order from the surface to the core (Figure 4A1). As mention above, GFAP-positive neural progenitors originate and migrate from subventricular zone via the rostral migratory stream (Lois and Alvarez-Buylla, 1994). The YFP-positive cells, therefore, may be not only astrocytes but also neurons in GFAP^Cre^-Ai39 mice. We therefore compared GFAP immunoreactivity with YFP fluorescence (Figures S2A1–A3). The GL, Mi, IPL and GrL were labeled strongly with GFAP antibody (Figure S2A2), but the immunoreaction in the EPL was weak (Figure S2A3). The labeling difference in the EPL suggested that the YFP fluorescence in this layer might be of the apical dendrites of inhibitory neurons in the GrL. Based on this finding, we considered that GFAP-AS was primarily distributed in the GL and IPL of the OB (Table 2). High densities of Olig2- AS were detected in the GL, EPL, and IPL (Figure 4B1).

#### Cerebral cortex

Olig2-AS were evenly distributed in most areas of the cerebral cortex. Among these areas, dense distribution was found in the somatosensory area (S) (Figures 4B2–B5), especially in the barrel field (S1BF) (Figures 4B4,B5). In addition, it was also conspicuous in the prefrontal cortex (PFC) (Figure 4B2), motor area (Figures 4B2–B5), retrosplenial area (RS) (Figures 4B5, 5B1–B3), visual area (Figures 5B1–B3). GFAP-AS were evenly distributed in almost all the cortical areas (Figures 4C, 5C).

As for the cortical layers, Olig2-AS are densely distributed in the layers 2–5, but are sparse in the layers 1 and 6 (Figures S2C1,C2). In contrast to the Olig2-AS distribution, GFAP-AS are preferentially localized to the sub-pial region (layer 1) and form the glia-limitans in all cortical areas (Figures 4C2–C5, 5C1–C3, Figures S2C3,C4).

#### Striatum and pallidum

We barely observed Olig2-AS in caudate-putamen (CPu) (Figures 4B3–B5) and accumbens nucleus (Acb) (Figure 4B3). In contrast, the lateral septal nucleus (LS) contained a high density of Olig2-AS (Figures 4B3,B4). GFAP-AS also accumulated densely in the LS and moderately in the CPu and Acb (Figure 4C3).

In the ventral pallidum (VP) area including the substantia innominata (SI) and the VP, a high density of Olig2-AS was observed (Figures 4B3,B4). As a result, these cells formed clear ventral borders of the CPu and Acb (Figures 4B3,B4). In the medial pallidum area including the medial septum nucleus (MS) and the diagonal band nucleus (NDB), Olig2-AS were also densely distributed (Figure 4B3). The NDB consists of an anteriorly located vertical limb (VDB), which branches posteriorly into separate bilateral horizontal limbs (HDB) (Herman et al., 2016). The high density of Olig2-AS distribution continued to the posterior HDB (Figure 4B4). In contrast, the density of GFAP-AS was low in the NDB (Figure 4C4) and the VP (Figures 4C3,C4). The globus pallidus is also known to belong to the dorsal pallidum, and consists of the LGP and the medial globus pallidus (MGP). Olig2-AS distribution was at a very high density in the LGP (Figure 4B5), and GFAP-AS density was also high in this nucleus (Figure 4C4). As mentioned above, however, GFAP-immunoreactive cells constitute a distinct population from Olig2-AS (Figures 2A,B, 3).

#### Thalamus and hypothalamus

In most thalamic nuclei, the density of Olig2-AS was very high (Figures 4B5, 5B1,B2), and that of GFAP-AS was generally low except anterodorsal and anteroventral thalamic nuclei (AD and AV, respectively) (Figures 4C5, 5C1,C2). In an exceptional case, the medial habenular nucleus (MHb) showed an opposite pattern; a low density of Olig2-AS and high density of GFAP-AS were detected (Figures 5B1,C1, Figure S2D).

In the hypothalamus, both Olig2-AS and GFAP-AS were distributed in high or moderate densities (Figures 4B4,B5,C4,C5, 5B1,C1). It should be noted that GFAP-AS was scarce in the subthalamic nucleus (STh) (Figure 5C1) and the zona incerta (ZI) (Figure 5C2), while Olig2-AS was densely distributed in these nuclei (Figures 5B1,B2).

#### Hippocampus

Olig2-AS were localized in the CA3 region and in the polymorph layer of the dentate gyrus of the hippocampus (Figures 5B1–B3). In the CA1 and CA2 regions, Olig2-AS distribution was limited to the pyramidal cell layer. In contrast, GFAP-YFP expression in GFAP^Cre^-Ai39 mice was confined to the molecular layer of the dentate gyrus (Figures 5C1–C3). It is well known that GFAP-positive neural stem cells generate neuroblasts and that the neuroblasts migrate to the granule layer, mature to become neurons and extend processes to the molecular layer (Lois and Alvarez-Buylla, 1994; Eriksson et al., 1998). Taking this into account, it is likely that GFAP-YFP signals found in the molecular layer was derived from neurons, but not from astrocytes. GFAP immunofluorescence in the hippocampus showed that GFAP-positive astrocytes were sparse in the molecular layer, discrepant from strong GFAP-YFP expression (Figure S2B). For this reason, we mapped distribution of GFAP-immunolabeled astrocytes, but not of GFAP-AS in the hippocampus (Table 2).

#### Midbrain and pons

Olig2-AS were found at high density in most of the midbrain nuclei, but, in contrast, GFAP-AS were scarce (Figures 5B3–C3). Both types of astrocytes were, exceptionally, co-localized in the SN. However, as in the case of LGP (see above), Olig2-AS did not co-express GFAP immunoreactivity (Figures 2C,D).

In the pons, Olig2-AS were found at high density, especially in the principal sensory trigeminal nucleus (Pr5), motor trigeminal nucleus (Mo5) and olivary/periolivary nuclei (LSO, SPO, DPO, and MVPO) (Figure 5B4). In contrast, GFAP-AS were barely observed in these areas (Figure 5C4).

#### Medulla and cerebellum

Olig2-AS were abundant throughout medulla nuclei. In particular, Olig2-AS were densely distributed in the spinal trigeminal nucleus (Sp5), facial nucleus (7N), and vestibular nuclei (VeN) (Figure 5B5). In contrast, GFAP-AS were observed at very low density in these regions. However, we observed strong expression of GFAP-YFP exceptionally in the cerebellar cortex, probably arising from the Bergmann glia (Figure 5C5).

In summary, the present mapping of two populations of astrocytes indicated a strong tendency for Olig2-AS and GFAP-AS to occupy different territories in the adult brain. Olig2-AS thus constitute a subpopulation of mature astrocytes and their distribution pattern implies functions specific to the various brain regions.

### Olig2-AS showed very similar distribution pattern to GAT3, but not to glutamate transporters, GLT-1/GLAST

**Figure 6:**
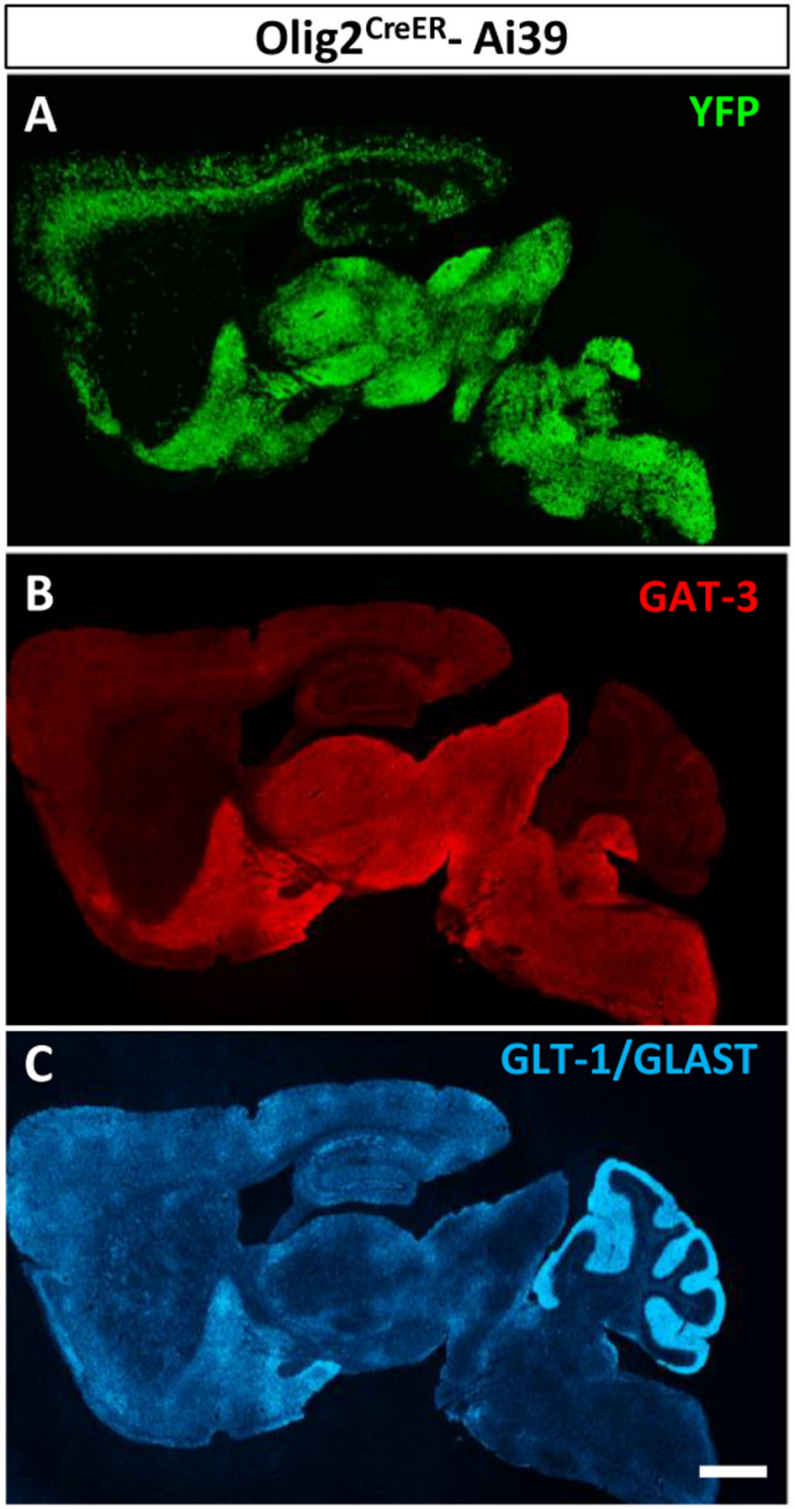
Olig2-AS distribution is similar to that of GAT-3, a GABA transporter. Low-magnification views of sagittal brain sections showed that the distribution of Olig2-AS **(A)** is similar to that of GAT-3 immunoreactivity, a glial GABA transporter **(B)**, but not to that of glutamate transporters, GLT-1 and /or GLAST **(C)**. Scale bar: 1 mm **(C)** for **A,B**).

We reported previously that Olig2-AS expressed GAT-3 in the globus pallidus and the cerebral cortex (Tatsumi et al., 2016). In this study, we noticed that the whole brain distribution map of Olig2-AS was very similar to that of GAT-3 (Figures 6A,B, respectively). On the other hand, the glutamate transporters, GLT-1/GLAST distributions were much less overlapped with those of Olig2-AS (Figures 6A,C).

### Fine processes of Olig2-AS were preferentially associated with GABAergic terminals in the LGP

**Figure 7:**
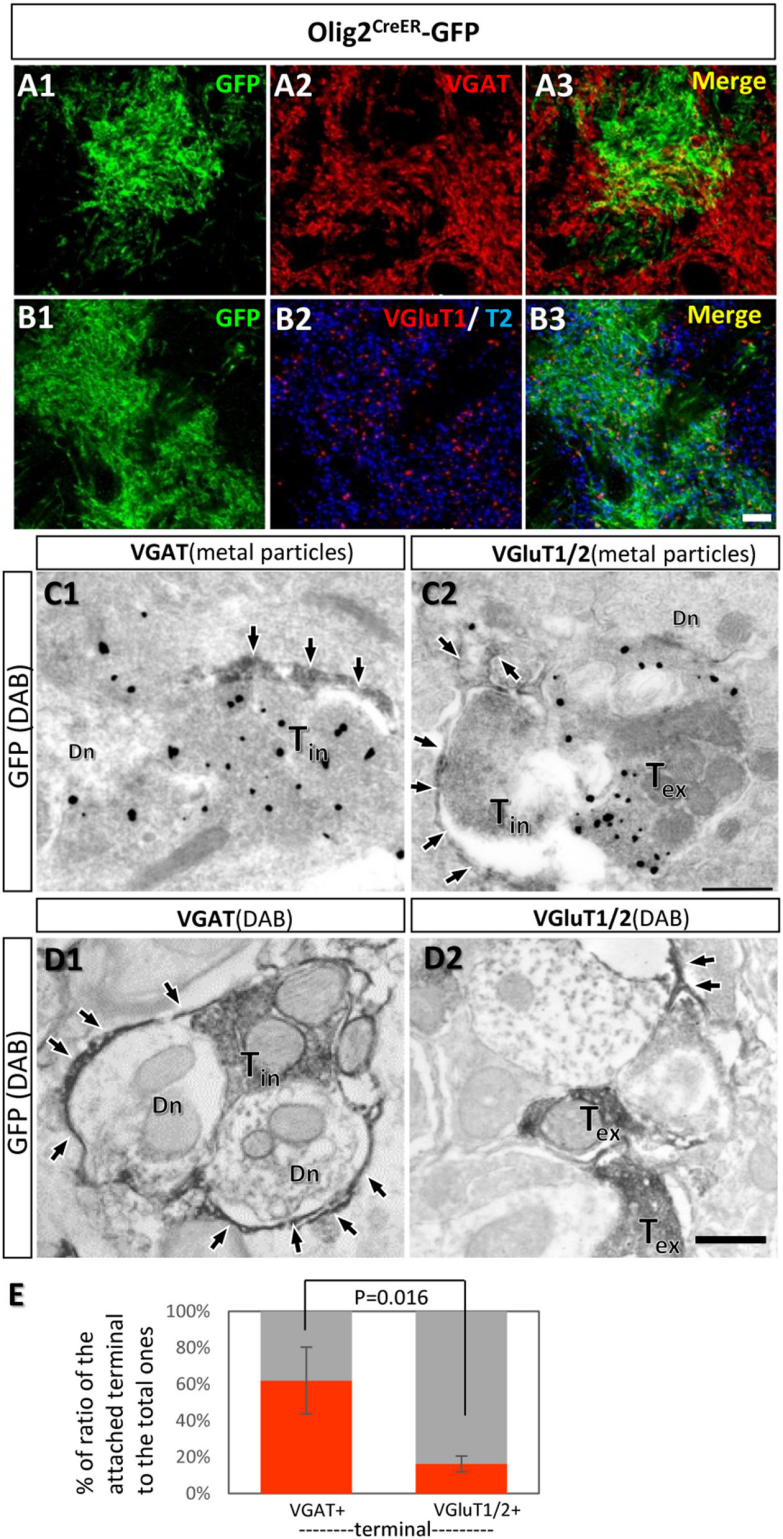
Fine processes of the Olig2-AS are closely associated with inhibitory terminals, but not with excitatory ones. **(A,B)** Low-magnification views of sections double-labeled with GFP and vesicular GABA transporter (VGAT) antibodies **(A1,A2)**, with GFP and vesicular glutamate transporter (VGluT1/2) antibodies **(B1,B2)** in the LGP of Olig2^CreER^-GFP mice. The GFP immunoreaction overlapped with VGAT and VGluT1/2 immunoreactivity **(A3,B3)**. **(C)** Double immunoelectron microscopic images with DAB reaction for GFP antibody and metal particle labeling for VGAT **(C1)** and VGluT1/2 **(C2)**. GFP-immunoreactive Olig2-astrocytic processes (arrows) made contact with VGAT-immunoreactive axon terminals (**C1**, T_in_). Olig2-astrocytic processes attached to an inhibitory terminal rather than metal particle-labeled excitatory terminal (**C2**, T_ex_). **(D)** Double immunoelectron microscopic images with DAB reactions for GFP antibody and for VGAT antibody **(D1)** or for VGluT1/2 antibodies **(D2)**. Olig2-astrocytic processes (arrows) made contact preferentially to inhibitory terminals with VGAT-immunoreactivity. **(E)** Semi-quantitative analyses of axon terminals (inhibitory and excitatory) with or without contacts of GFP-positive astrocytic processes are shown. The percent ratio of the inhibitory terminals with process attachments to the total inhibitory terminals was 61.9 ± 18.4% (n = 3, attached terminals/total terminals; 15/37, 10/21, 29/37), while the ratio of the excitatory terminals with process attachments to the total excitatory terminals was 16.2 ± 4.39% (*n* = 3, attached terminals /total terminals; 9/46, 5/50, 4/21). The difference of the ratios was statistically significant (*p* = 0.016, one-way ANOVA with *post-hoc* tukey HSD test). T_in_, inhibitory terminal; T_ex_, excitatory terminal; Dn, dendrite; Scale bar: 10 μm (**B3**, for **A1,B2**), 500 nm (**D2**, for **C1,D1**).

The transporter expression patterns prompted us to examine the spatial relationships between fine processes of Olig2-AS and axon terminals of inhibitory or excitatory neurons. The double immunofluorescence experiments using anti-GFP antibody and anti-vesicular GABA transporter (VGAT) antibody or anti-vesicular glutamate transporters (VGluT1/2) antibodies were performed in the LGP of Olig2^CreER^ -GFP mice (Figures 7A,B). The reason we chose the LGP is that the nucleus receives glutamatergic inputs from the subthalamic nucleus in addition to dense GABAergic inputs from the striatum (Kita and Kitai, 1987). We observed the strong expression of VGAT (Figure 7A2) and the moderate expression of VGluT1/T2 in the LGP (Figure 7B2) and Olig2-AS overlapped with both VGAT and VGluT1/2 immunoreactivities in the low magnification views (Figures 7A3,B3). At this light microscopic level, Olig2-AS appeared to be associated with both excitatory and inhibitory terminals, but the actual associations should be verified at an electron microscopic level. We therefore performed double immunoelectron microscopy with GFP and VGAT or VGluT1/2 antibodies in the Olig2^CreER^-GFP mice. We found that GFP-immunoreactive astrocytic processes (arrows, Figures 7C1,C2) tended to be associated with VGAT metal particle-labeled axon terminals (Figure 7C1, T_in_), but not with VGluT1/2 metal particle-positive terminals (Figure 7C2, T_ex_). When we changed the labeling methods to the opposite way (axon terminals were labeled with GFP and astrocytic processes with metal particles), similar tendency was observed (Supplementary Figures S3A,B). We further observed the preferential association of astrocytic processes with GABAergic terminals in the cases that both astrocytic and neuronal elements were double-labeled with DAB (Figures 7D1,D2, Supplementary Figures S3C,D). Semi-quantitative analyses revealed that 56.8% (54/95, *n* = 3) of VGAT-immunoreactive terminals made contact with fine processes of Olig2-AS, while 15.4% (18/117, *n* = 3) of VGluT1/2 immunoreactive terminals did so (Figure 7E).

## Discussion

Astrocytes are the most abundant glia cell type in the central nervous system (CNS) and they perform diverse functions in the brain. Accumulating evidence has revealed that astrocytes modulate synaptic activities by promoting neurotransmitter uptake from synaptic clefts and/or by releasing so-called gliotransmitters such as glutamate, D-serine, and ATP into synaptic clefts (Volterra and Meldolesi, 2005; Welberg, 2009). This type of modulation takes place in various brain regions and the concept of the tripartite synapse, consisting of pre- and post-synaptic neurons and astrocytic fine processes, has emerged and become widely accepted (Perea et al., 2009). In addition to the regulation of neurotransmission, astrocytic fine processes are implicated in synapse formation (Chung et al., 2015) and in formation of blood-brain barriers (Abbott et al., 2006). Such a variety of astrocytic functions suggests that there exist subtypes of astrocytes that play different roles. In line with this notion, the idea of astrocyte heterogeneity has attracted considerable interest (Bayraktar et al., 2015; Schitine et al., 2015). However, it is still an open question whether there are subpopulations specific for certain functions.

Cajal first divided astrocytes into two groups: fibrous astrocytes and protoplasmic astrocytes (Ramón y Cajal, 1897). This morphological classification corresponds to regional heterogeneity, because fibrous and protoplasmic astrocytes generally segregate in white matter and gray matter, respectively. Recent studies demonstrated that astrocytes are allocated to spatially distinct domains in the developing brain (Tsai et al., 2012; Molofsky et al., 2014; Bayraktar et al., 2015) and can only interact with neurons derived from the same domain (Torigoe et al., 2015). Given that astrocytic regional domains coordinate post-natal neural circuit formation and regulate eventual neuronal activity, astrocytic regional heterogeneity can also be regarded as functional heterogeneity. In our previous study, we reported that a subpopulation of astrocytes expressed a specific type of chondroitin sulfate proteoglycan (CSPG) recognized by CS56 monoclonal antibody (Hayashi et al., 2007). We identified the CSPG as tenascin-R (TNR) and the astrocytic population was distributed in the cerebral cortex of the various species, including human, primate, domestic pig and rodents. We further demonstrated that TNR regulated the expression of GLAST, a glutamate transporter, suggesting that the subset of astrocytes might be involved in the glutamate homeostasis in the cerebral cortex (Okuda et al., 2014).

With regard to regional heterogeneity, fluorescence-activated cell sorting (FACS) was recently applied to astrocytic classification (Zamanian et al., 2012; John Lin et al., 2017; Sun et al., 2017). John Lin et al. identified five distinct subpopulations from Aldh1l1-GFP transgenic adult mice (John Lin et al., 2017). They collected astrocytes from five different brain regions and compared the distribution of these subpopulations. They found that the most abundant subpopulation existed evenly in all regions and expressed synapse-related genes specifically, while another subpopulation clearly localized in certain specific regions. Interestingly, these subpopulations participated in the onset or progression of malignant glioma. These findings suggested that diverse astrocytic functions are provided by subpopulations not only under physiological conditions but also in the injury-disease state. Zamanian et al. profiled gene expression patterns in reactive astrocytes from Aldh1l1-GFP transgenic adult mice using the FACS technique (Zamanian et al., 2012). They showed that reactive astrocytes were highly heterogeneous in terms of gene expression patterns, and their transcriptome database generated the idea that there are broadly two subtypes of reactive astrocytes; furthermore, these two types were recently termed A1 and A2 astrocytes reminiscent of M1/M2 macrophages (Liddelow et al., 2017). The study demonstrated that A1 astrocytes were harmful for the surrounding microenvironment, and, on the contrary, that A2 astrocytes were beneficial for injured neuronal cells. Taken together, these recent reports strongly suggest that astrocytic subpopulations have differential roles in both physiological and pathological states.

In the present study, we suggest that the transcription factor Olig2 is involved in the formation of a subpopulation of astrocytes (Olig2-AS) in the adult brain. Olig2-AS have a distinct distribution pattern and express very low level of GFAP protein, although they express mature astrocytic markers such as S100β, GS, and 3-PGDH (Yamasaki et al., 2001). Immunoelectron microscopy demonstrated that Olig2-AS formed endfeet that were attached to brain capillaries (data not shown), also suggesting that Olig2-AS form blood-brain barrier, an important astrocytic function. We assume that astrocytes detected in other transgenic lines such as ALDH1L1-GFP mice also exert similar functions (Zamanian et al., 2012).

So far, several different types of GFAP-transgenic lines have been established (Brenner et al., 1994; Zhuo et al., 1997; Nolte et al., 2001; Casper and McCarthy, 2006; Lee et al., 2006). But the expression patterns of reporter proteins, for example GFP or lacZ, are not always consistent among these strains. GFAP^Cre^ mice we employed in the present study allowed detection of GFAP-expressing cells in a number of brain regions including subventricular zone (Imura et al., 2003). Neuronal stem cells are known to express GFAP (Kempermann et al., 1997; Eriksson et al., 1998; Seri et al., 2001) and in our GFAP immunofluorescence we indeed observed GFAP-immunoreactive neuronal processes in the EPL layer of the OB (Figure 2A2). Such different patterns of GFAP genetic labeling prompted a caution that the GFAP-AS detected in the present study may not cover all the GFAP-positive astrocytes. Interestingly, at least in the LGE and the SN, Olig2 genetic labeling combined with GFAP immunofluorescence demonstrated that two populations (Olig2-AS and GFAP-positive astrocytes) exhibited mutually-exclusive patterns of distributions (Figure 3). The reason why Olig2-AS expressed low level of GFAP is an open question.

In our previous report, we suggested that Olig2 astrocytes are subsidiary to inhibitory GABAergic transmission (Tatsumi et al., 2016). The present mapping of Olig2-AS in the adult brain and comparison with that of astrocytic GABA transporter reinforced this idea. The current members of GABA transporter family are GAT-1, -2, and -3. GAT-1 is primarily expressed in GABAergic neurons and is involved in GABA uptake from synaptic clefts. GAT-2 and -3 are of glial subtypes and GAT-3 is the dominant type between two (Zhou and Danbolt, 2013). Interestingly, a recent report demonstrated that astrocytes could release GABA by the reverse action of GAT-2 or -3 (Héja et al., 2012). Olig2-AS may also exert inhibitory functions with the reverse action of GAT-3. We further demonstrated that fine processes of Olig2-AS preferentially made contact with terminals of VGAT-positive axons rather than VGluT1/2-positive excitatory ones in the LGP. A caveat to this result is that the LGP receives more inhibitory inputs than excitatory ones (see Figures 7A2,B2) and the chance of astrocytic contacts to the excitatory terminals might be hampered. However, in the present study, we chose the territories of Olig2-AS, where the GFP-positive fine processes are enriched. In these fields, we picked up comparable numbers of inhibitory terminals (total number 95 from three animals) and excitatory terminals (total number 117 from three animals), but we detected significant difference in the numbers of astrocytic process-attached terminals (inhibitory; 54 out of 95, excitatory; 18 out of 117). The difference can contradict the caveat. We do not know whether the preferential contacts of Olig2-AS to inhibitory terminals can be observed in other brain regions and what kind of astrocytes compensate for the lack of contacts to excitatory terminals. These are intriguing questions for future studies. The morphological observation under an electron microscope provides strong circumstantial evidence and it is tempting to speculate that there may be a subpopulation of astrocytes that is specific to certain neurotransmitters. This kind of heterogeneity may be barely detectable if one isolates and compares astrocytes from two or more brain regions (Zamanian et al., 2012; John Lin et al., 2017). The hypothesis appears to be compatible with the idea that astrocytes and neurons derived from the same domain of the embryonic brain can only interact each with each other (Torigoe et al., 2015), because GABAergic interneurons arise from Olig2-expressing precursors (Miyoshi et al., 2007). However, whether Olig2-AS in the adult brain really arise from Olig2-positive precursors and regulate GABA transmission awaits further studies. The developmental origin and lineage tracing of Olig2-AS are thus critical in providing an answer to the hypothesis and are currently under investigation.

## Author contributions

KT designed research; KT, AI, MY, SM-T, YK, KN, YT, TS, HO, and TT performed research; KT and AW analyzed data and wrote the paper.

### Conflict of interest statement

The authors declare that the research was conducted in the absence of any commercial or financial relationships that could be construed as a potential conflict of interest.

## Abbreviations

3-PGDH: rabbit anti-3-phosphoglycerate dehydrogenase
MS: medial septum nucleus
7N: facial nucleus
Mve: medial vestibular nucleus
Acb: accumbens nucleus
NDB: diagonal band nucleus
AD: anterodorsal thalamic nucleus
OPT: olivary pretectal nucleus
AV: anteroventral thalamic nucleus
PaPo: paraventricular hypothalamic nucleus, posterior part
AH: anterior hypothalamic area
PaV: paraventricular hypothalamic nucleus, ventral part
AM: anteromedial thalamic nucleus
PF: parafascicular thalamic nucleus
APT: anterior pretectal nucleus
PFC: prefrontal cortex
BMP: basomedial amygdaloid nucleusposterior part
PH: posterior hypothalamic area
BST: bed nucleus of the stria terminals
Pir: piriform cortex
CbX: cerebellar cortex
PMCo: posterior cortical amygaloid nucleus
Cg/RS: cingulate retrosplenial cortex
Pn: Pontine nuclei
CM: central medial thalamic nucleus
Po: posterior thalamic nuclear group
Cpu: caudla putamen
PPT: posterior pretectal nucleus
DCN: deep cerebellar nuclei Pr5 principal sensory trigeminal nucleus
DG: dentate gyrus
PV: paraventricular thalamic nucleus
DpMe: deep mesencephalic nucleus
RSA: retrosplenial agranular cortex
EPL: external plexiform layer
RSG: retrosplenial granular
GL: glomerular laye
Rt: reticular thalamic nucleus
GrDG: dentate gyrus, granule cell layer
S: somatosensory cortex
GrL: granular cell layer
S100β: S100 calcium-binding protein B
GS: glutamine synthetase
S1BF: primary somatosensory cortex, barrel field
HY: Hypothalamus
SC: superior colliculus
IC: inferior colliculus
SCh: suprachiasmatic nucleus
LGP: lateral globus pallidus
SI: substantia innominate
LH: lateral hypothalamic nucleus
SN: substantia nigra
LHb: lateral habenular nucleus
Son: superior olivary nuclei
LP: lateral thalamic nucleuar group
Sp5: spinal trigeminal nucleus
LPO: lateral preoptic area
Spa: subparaventricular zone of the hypothalamus
LSD: lateral septal nucleus, dorsal part
SpVe: spinal vestibular nucleus
LSI: lateral septal nucleus, intermediate
STh: subthalamic nucleus
LV: lateral ventricle
SuVe: superior vestibular nucleus
M: motor cortex
Th: Thalamus
MD: mediodorsal thalamic nucleus
V1, V2: visual cortex
MG: medial geniculate nucleus
VA: ventral anterior thalamic nucleus
MG: medial geniculate nucleus
VeCb: vestibulocerebellar nucleus
MHb: medial habenular nucleus
vGluT1: vesicular glutamine transporteranti-1
Mi: mitral cell layer
vGluT2: vesicular glutamine transporteranti-2
MM: Mammillary body
VLG: ventral lateral geniculate nucleus
Mo5: motor trigeminal nucleus
VP: Ventral pallidun
MPA: medial preoptic area
VPL: ventral posterolateral thalamic nucleus
MPO: medial preoptic nucleus
VPM: ventral posteromedial thalamic nucleus
MPT: medial pretectal nucleus
ZI: zona incerta.
