# Supplementary material for: Olig2-Lineage Astrocytes: A Distinct Subtype of Astrocytes That Differs from GFAP Astrocytes
Source: Front Neuroanat. 2018 Feb 14;12:8. doi: 10.3389/fnana.2018.00008 (PMC5819569; doi:10.3389/fnana.2018.00008)
Supplement: Supplementary file 1 [file Image1.pdf]

Supplementary Fig.S1

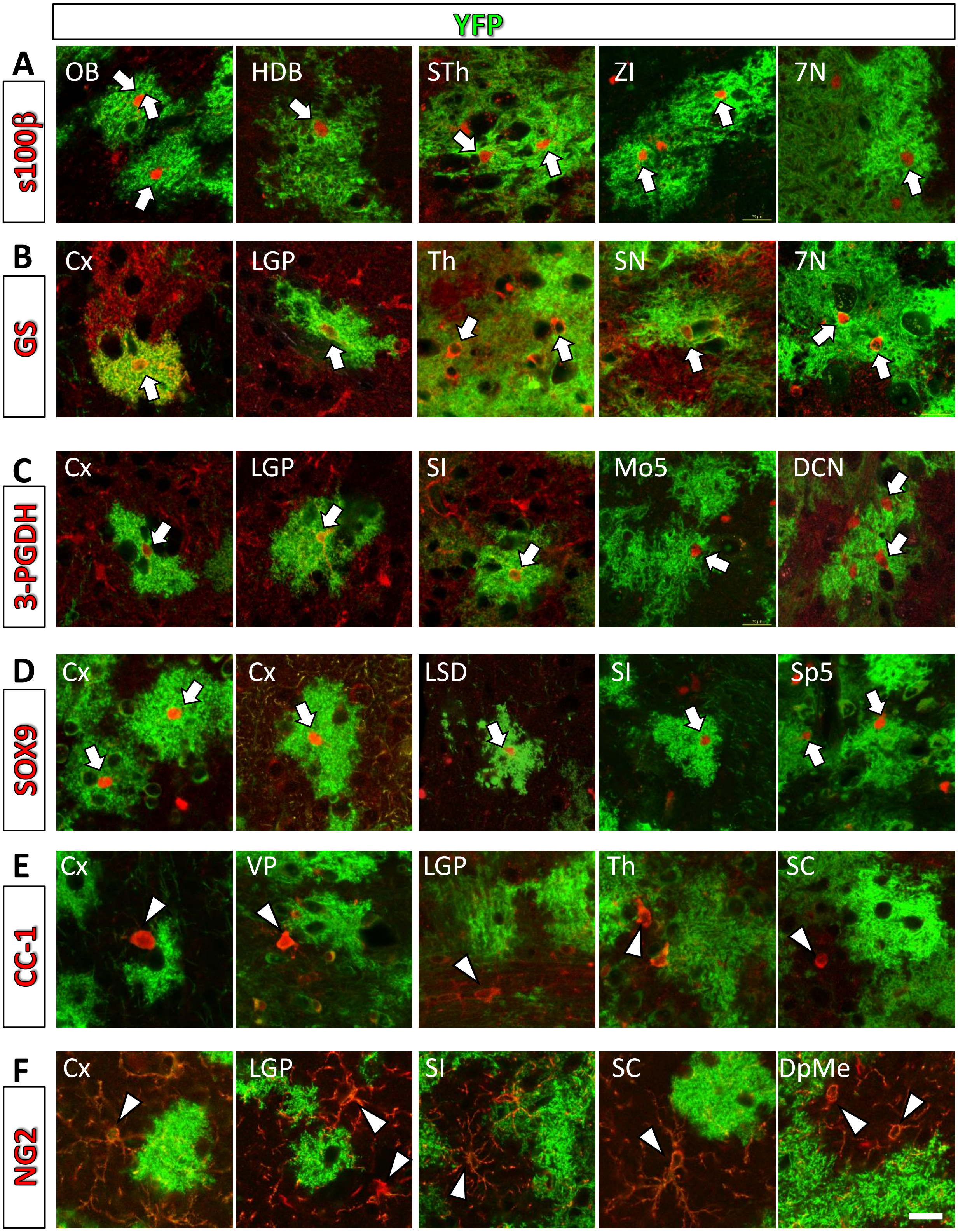

Supplementary Figure S1:

Olig2-lineage bushy type cells co-expressed several astrocyte markers; s100β (**A**), glutamine synthetas (GS) (**B**), 3-phosphoglycerate dehydrogenase (3PGDH) (**C**), SOX9 (**D**) in their nuclei (arrows). But they were negative for mature oligodendrocyte marker, CC-1(**E**) and NG2 (**F**) (arrowheads) .

OB = olfactory bulb, HDB = diagonal band horizontal limb, STh = subthalamic nucleus, ZI = zona incerta, 7N = facial nucleus, Cx = cortex, LGP = lateral globus pallidus, Th = thalamus, SN = substantia nigra, Mo5 = motor trigeminal nucleus, DCN = deep cerebellar nuclei, LSD = lateral septal nucleus, dorsal part, SI = substantia innominata, Sp5 = spinal trigeminal nucleus, VP = ventral pallidum, , SC = superior colliculus, DpMe = deep mesencephalic nucleus

Scale bar: 20 μm.

Supplementary Fig.S2

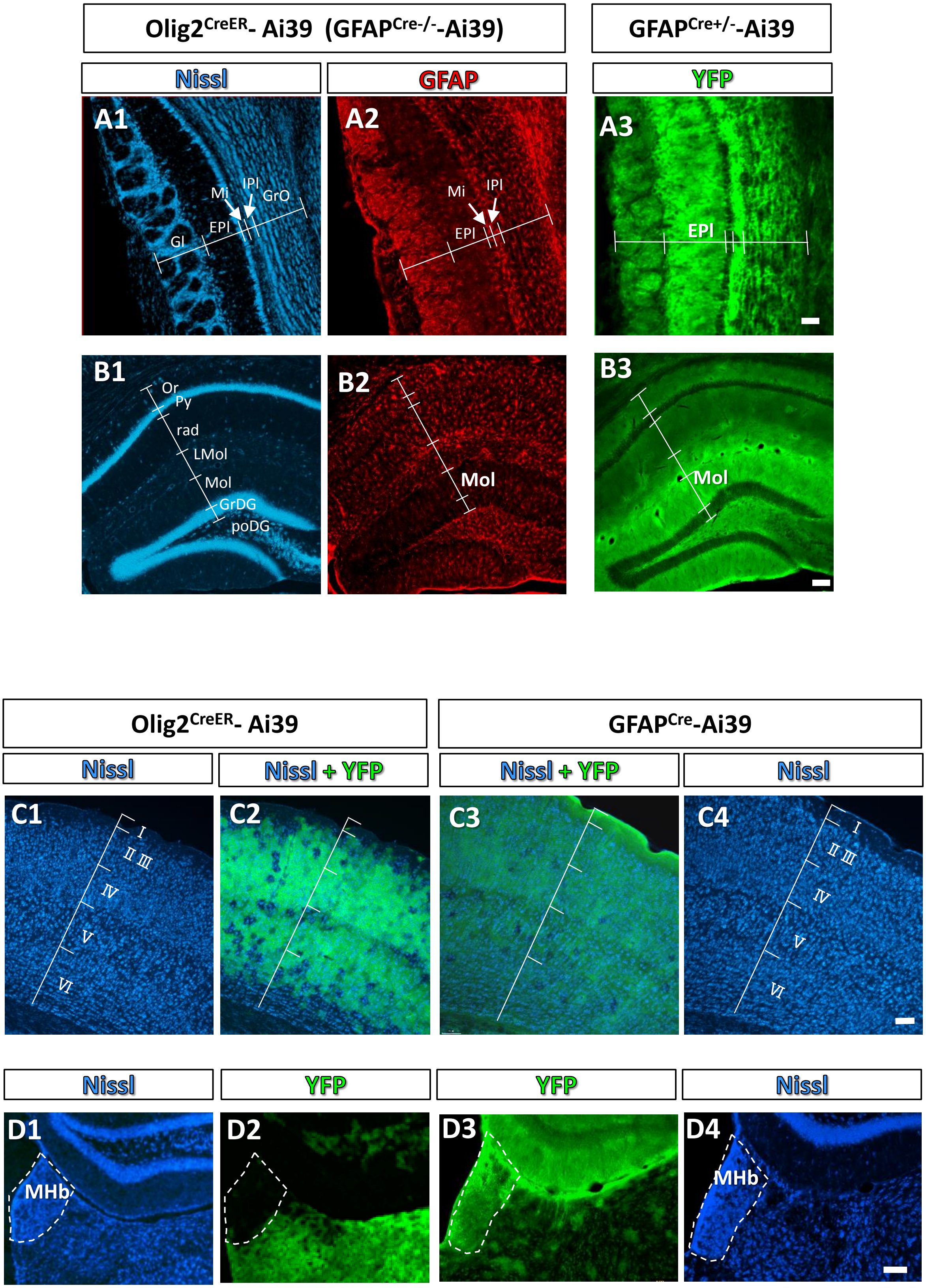

Supplementary Figure S2:

(**A-B**) GFAP immunoreactive cells and GFAP-lineage YFP cells are not always consistent. GFAP immunostaining was performed to the section of Olig2^{CreER}-Ai39 mice (GFAP$^{Cre-/-}$) with Nissl counter-staining (**A1-A2, B1-B2**). GFAP immunoreaction patterns in OB and Hippocampus are different from that of YFP expression in the GFAP$^{Cre+/-}$-Ai39 (**A3, B3**). For example, the external plexiform layer (EPL) in the OB and molecular layer of dentate gyrus (Mol) show weak GFAP immunoreaction (**A2** and **B2**) ,but YFP intensity are strong in these layers (**A3** and **B3**).

(C) The expression pattern of YFP in cortical layers (S1BF) of Olig2^{CreER}-and GFAP$^{Cre+/-}$-Ai39 mice are shown. The Olig2-AS are density in layers 2-5, but are scarce in layers1 and 6. In contrast, GFAP-AS are preferentially localized to the sub-pial region (layer 1) and form the glia-limitans in all cortical areas. (**D**) The distribution patterns are reversed in the medial habenular nucleus (MHb) between the Olig2^{CreER}-and GFAP$^{Cre+/-}$-Ai39 mice.

Scale: 200 mm (**A, B**) 100 mm (**C, D**)

Supplementary Fig.S3

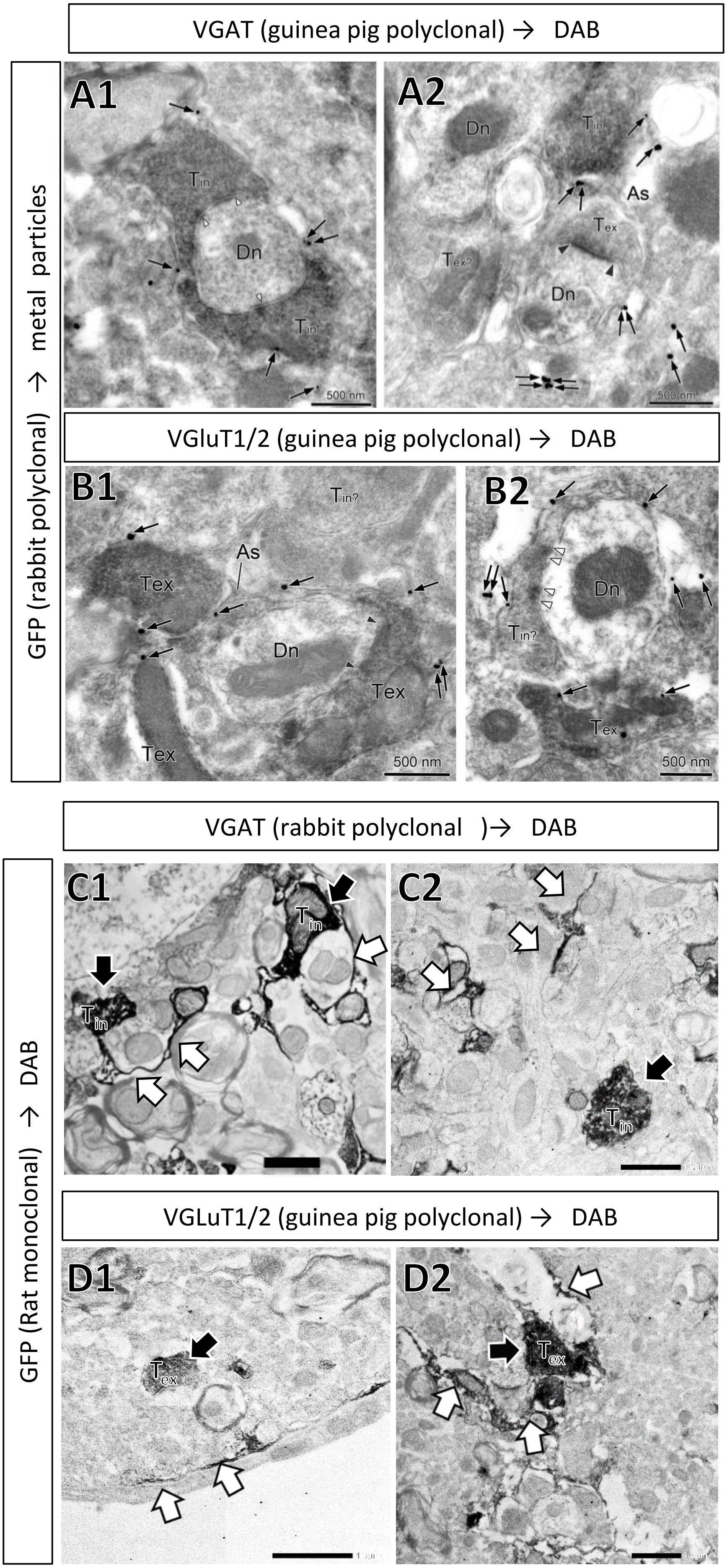

Supplementary Figure S3:

The double immunoelectron microscopy with GFP and VGAT or VGluT1/2 antibodies in the Olig2^{CreER}-GFP mice.

The metal particles -labeled astrocyte processes (**A1-B2**, arrows) tended to be associated with VGAT DAB-labeled axon terminal (**A1-A2**, $T_{in,}$), but some processes left a space and did not contact (**A2**). We rarely observed the contact metal particles -labeled astrocyte processes and VGluT1/2 terminals (**B1-B2**, T_{ex}). This tendency was also observed in the case of the GFP- immunoreactive astrocyte processes (**C1-D2**, arrows) and DAB labeled inhibitory (**C1-C2**, T_{in}) or excitatory terminals (**D1-D2**, T_{ex}). Dn: dendrite. Scale: 500 nm (**A, B**) 1 μm (**C, D**)
